# Polyoxometalate–peptide hybrid materials: from structure–property relationships to applications

**DOI:** 10.1039/d2sc05105b

**Published:** 2022-11-16

**Authors:** Héctor Soria-Carrera, Elena Atrián-Blasco, Rafael Martín-Rapún, Scott G. Mitchell

**Affiliations:** a Instituto de Nanociencia y Materiales de Aragón (INMA), CSIC-Universidad de Zaragoza c/ Pedro Cerbuna 12 50009 Zaragoza Spain rmartin@unizar.es scott.mitchell@csic.es; b CIBER de Bioingeniería, Biomateriales y Nanomedicina, Instituto de Salud Carlos III 28029 Madrid Spain; c Departamento de Química Orgánica, Facultad de Ciencias, Universidad de Zaragoza c/ Pedro Cerbuna 12 50009 Zaragoza Spain

## Abstract

Organo-functionalisation of polyoxometalates (POMs) represents an effective approach to obtain diverse arrays of functional structures and materials, where the introduction of organic moieties into the POM molecules can dramatically change their surface chemistry, charge, polarity, and redox properties. The synergistic combination of POMs and peptides, which perform a myriad of essential roles within cellular biochemistry, including protection and transport in living organisms, leads to functional hybrid materials with unique properties. In this Perspective article, we present the principal synthetic routes to prepare and characterise POM–peptide hybrids, together with a comprehensive description of how their properties – such as redox chemistry, stereochemistry and supramolecular self-assembly – give rise to materials with relevant catalytic, adhesive, and biomedical applications. By presenting the state-of-the-art of the POM–peptide field, we show specifically how emerging chemical approaches can be harnessed to develop tailored POM–peptide materials with synergistic properties for applications in a variety of disciplines.

## Abstract 2

Organo-functionalisation of polyoxometalates with peptide moieties is an effective approach to obtain diverse arrays of functional hybrid materials, where each component influences the resulting change, surface chemistry, polarity, and redox properties.

## Graphical abstract

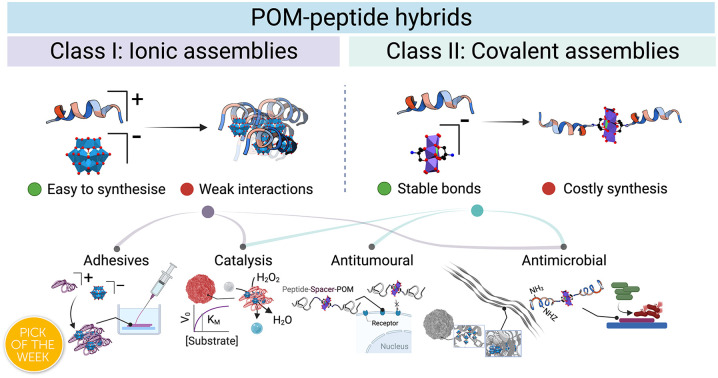

## 1. Introduction

Transition metals play key roles in biological processes. In Nature, the chemistry of polyoxometalates (POMs) – anionic metal-oxo clusters – and peptides has played a crucial role in metabolism. The clearest example is the molybdenum storage protein (MoSto) found in some N_2_-fixing bacteria that produce discrete polynuclear molybdenum-oxide clusters by biomineralisation.^1–3^ The MoSto protein uses ATP hydrolysis to ensure a high loading of Mo(vi) as structurally diverse polyoxomolybdate clusters fixed by polypeptides. This biomineralisation takes place even under Mo starvation conditions.

**Fig. 1:**
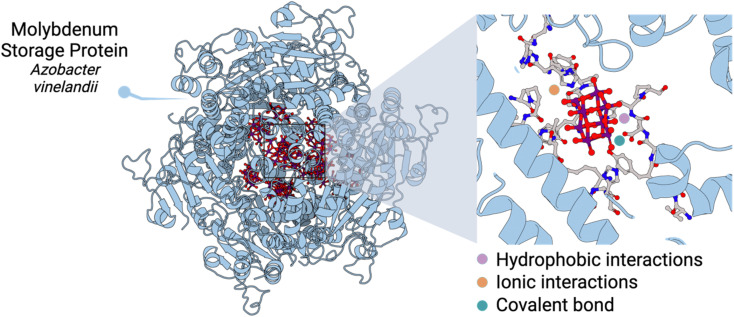
Structure of the molybdenum storage protein (MoSto) and insight into one catalytic centre containing a polyoxomolybdate anion.

At the molecular level, the different cavities present in MoSto template the biosynthesis of different POM archetypes which are both ionically or covalently linked to the protein. One key aspect is that POMs can interact with basic amino acid residues, form hydrogen bonds, and establish hydrophobic interactions depending on the location and type of amino acid residues nearby (Fig. 1). Crucially, the confined interior environment of the protein protects the POMs from the physiological medium in which they are no longer stable. Indeed, it is known that subtle changes in pH trigger Mo release, which can promote the hydrolysis and dissociation of POMs into MoO_4_^2−^.^3^

The capacity of POMs to interact and stabilise proteins is charge- and size-dependent.^4,5^ Moreover, POMs share similar hierarchical structural features and functionality with enzymes, Nature's biological catalysts. POMs, as enzymes, rely on their structure and redox activity to catalyse reactions efficiently. Yet, their activities are transient and pH-sensitive in aqueous solutions.

**Fig. 2:**
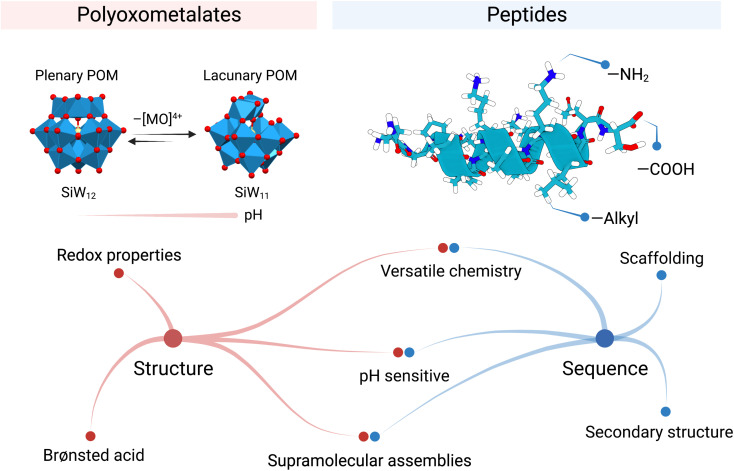
Example structures and properties of polyoxometalates and peptides.

The silicotungstate Keggin ion, {SiW_12_}, for example, undergoes multiple structural transformations when pH gradually rises.^6^ These interconversions alter the structural composition of the POM and in consequence modify its reactivity. The mono-lacunary Keggin ion, {SiW_11_}, is more reactive than {SiW_12_} and can coordinate metal ions^7^ or react with electrophiles like phosphonates or silicates (Fig. 2).^8^ Rompel and co-workers recently reviewed the speciation of multiple POMs with pH, highlighting the importance of these equilibria on POM stability. We encourage interested readers to refer to this article as a “Rosetta stone” when considering the true nature of the POM present in their system.^9^

POMs showcase important features that make them valuable catalysts in Industry. For example, a unique property associated with POMs is that, generally, their structure remains unaltered after electron transfer and storage. Plus, POMs are strong Brønsted acids, so they transfer protons both in solution and in the solid state where the resultant anionic cluster remains stable as well.^10^ These predictable electron and proton transference properties combined with their large structural diversity, combinatorial potential, and ability to be supported on a range of substrates, make POMs appealing and valuable catalysts.^11,12^

Another recent application of POMs relies on their use as additives in protein crystallisation. Herein, the anionic nature of POMs promotes crystal formation and packing, which facilitates structure elucidation.^13^ Protein crystallography is notoriously complex because of the intricate three-dimensional structural conformations of many proteins. This beneficial effect of POMs was demonstrated through the addition of a hetero-polyoxotungstate cluster to ribosome crystals resulting in the improved resolution of the structure determination.^14,15^ POMs contribute with their anomalous phasing power as well as stabilisers of selected functional conformations due to their electrostatic interactions with macromolecules.

The other building blocks this Perspective article discusses are peptides. Peptides are a class of biomolecules that are ubiquitous in biology and have important roles in catalysis, signalling, and transport, among others. Peptide chemistry is mainly governed by the unique amino acid sequence of the peptide, where the combination of chemical functional groups of the amino acids (*i.e.*, hydrophobic, basic, acidic, and polar uncharged) allows peptides to fold specifically (Fig. 2). Thus, peptide sequence determines both structure and function. For instance, the amino acid sequence of antimicrobial peptides (AMPs) can be decisive in specifically killing bacterial over mammalian cells due to the subtle differences in surface charge. Also, the active centres in proteins present cooperation between amino acids, which facilitates catalysis and increases specificity, *e.g.* the catalytic triad in trypsine. The sequence is also important when incorporating inorganic (metal ion) cofactors in the protein structure. Only a few amino acids coordinate the metal at a precise place. This marriage expands the possible chemical transformations, for instance, redox reactions.

Peptide folding is, in essence, a highly dynamic process which facilitates adaptation, a crucial aspect for Biology, though it is also a challenge when designing peptides. The presence of salts or chaotropic agents could lead to undesired folding that could have a detrimental effect on the predicted role. Moreover, the presence of proteolytic enzymes in the biological milieu could also disrupt the peptidic activity. Interestingly, Parac-Vogt's group has reported Zr- and Hf-based POMs that successfully catalyse peptide bond scission.^16,17^ To overcome peptide misfolding, numerous strategies have been reported to face these challenges like using conformationally restricted peptides^18^ or encapsulating them inside nanoparticles.^19^

Combining POMs with peptides represents a unique opportunity to produce hybrid materials with synergistic properties stemming from the inorganic and organic components. POMs and peptides can be combined in hybrid materials where the POMs trigger conformational changes leading to reinforced structures. As a result, several groups have already started to explore the opportunities offered by these hybrid materials, which has provided already insights into the interplay of POMs and peptides and also fascinating functional materials.

**Fig. 3:**
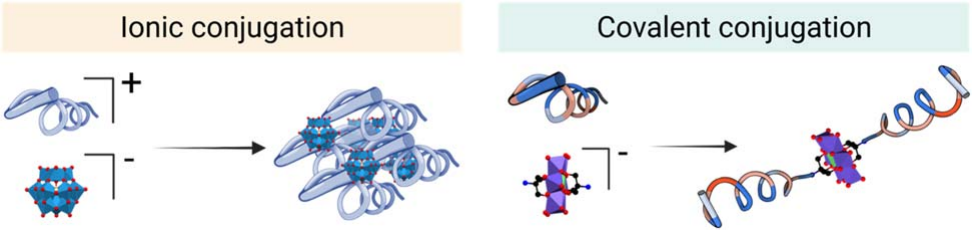
Formation of POM–peptide hybrids *via* ionic interaction (left) and covalent binding (right).

In the literature, POM–peptide hybrids have been classified according to the connectivity between the POM and the peptide, that is, whether the linkage is ionic (class I) or covalent (class II) (Fig. 3).^20^ Both synthetic strategies provide opportunities to develop synergistic materials that gather and improve the properties of the individual components.

On the one hand, the synthesis of class I hybrids represents a simple yet versatile synthetic approach to prepare materials from relatively simple and accessible POM and peptide building blocks. The critical parameters to consider in their design are the size and geometry of the components, as well as their stoichiometry.^21^ On the other hand, the covalent organo-functionalisation approach, involved in the preparation of class II hybrids, offers greater control over the physicochemical properties of the resulting materials and can be used to access truly functional molecules more accurately.

The increasing number of POM–peptides publications in recent years demonstrates a steadily growing sub-field in POM-hybrid research. One notable aspect is that many of the studies reported in the literature routinely report the application of these hybrid systems. For example, peptides provide confined environments that promote catalysis, thus the incorporation and encapsulation of POMs inside these pockets favours their catalytical properties.^22,23^ Both POMs^24^ and peptides^25^ can display antimicrobial properties, so it is not surprising that researchers are investigating the combination of both materials to yield high-performance antimicrobials. We^22,26^ and others^27,28^ have studied how POM–peptide systems interact with bacteria, yet there is still a lack of understanding of the underlying mechanisms behind the excellent antimicrobial properties.

In this Perspective review, by surveying the literature published to-date, we aim to provide insight into the structure–properties relationships of POM–peptide hybrids to provide a guide to researchers who require access to determined physicochemical properties in order to pursue a variety of subsequent applications. To do so, we will discuss the most relevant aspects of POM–peptide chemistry in the class I (ionic) and II (covalent) hybrid systems and show how structure–activity studies shed important new information relevant to their application as catalysts, adhesives, bioactive materials, and more.

## 2. Ionic POM–peptide hybrids

**Fig. 4:**
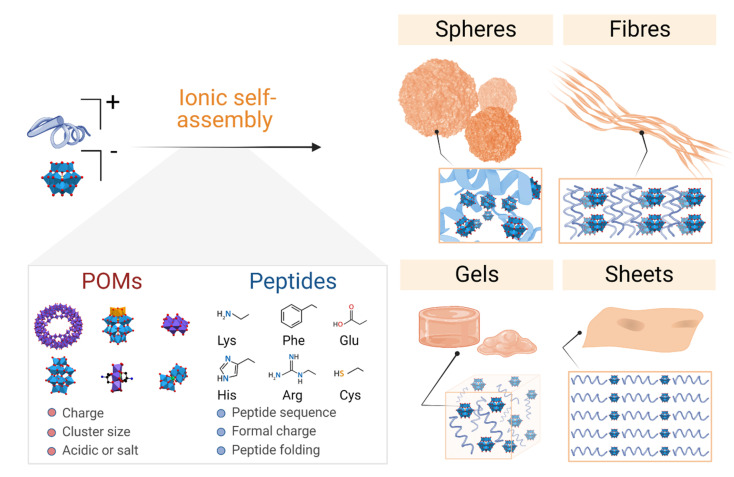
Overview of the main supramolecular structures and networks formed by bicomponent ionic POM–peptide hybrids and the most relevant properties of their precursors.

The literature on POM–peptide hybrid materials is dominated by the class I hybrids, which are those formed by electrostatic interactions between the POM and peptide components. Indeed, some examples from the current state-of-the-art have been addressed by recent review articles which cover the fundamentals of hybrid formation and their applications^20,21,29^ and the interaction of POMs with peptides and proteins.^21,29^ In this section we will discuss the different examples of ionic POM–peptide hybrids published so far, focusing on their macromolecular structures and the characteristics of the material preparation to achieve the different structures, from the starting materials to the assembly conditions and driving forces (Fig. 4).

### 2.1. Formation of binary hybrids: from their microscopic structures to their macromolecular properties

**Fig. 5:**
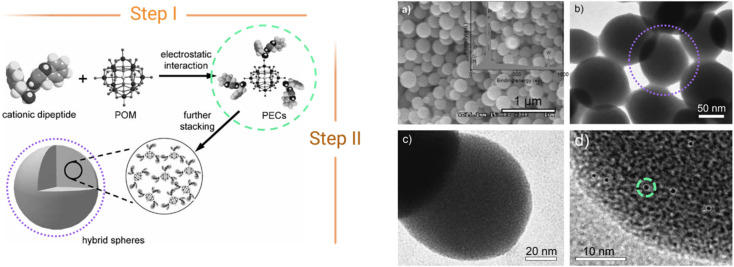
Scheme of the two-step self-assembly of ionic POM–peptide hybrids, (left) and nanospheres formed as observed by HR-TEM (right). Adapted from ref. 30 with permission John Wiley and Sons, copyright 2010.

The first reported ionic assembly of POMs and peptides for the creation of a new material was, to the best of our knowledge, the combination of {HSiW_12_} and a cationic dipeptide derived from diphenylalanine (FF) into hybrid colloidal spheres of *ca.* 150 nm.^30^ The self-assembly mechanism of the cationic peptide with the anionic POM follows two-steps (Fig. 5). In the first step, the main driving force for the self-assembly is the electrostatic interaction between the opposed charges of the components forming clusters in which the POM (negatively charged) is surrounded by encapsulating peptide (positively charged) units (Fig. 5, green circles). Hydrogen bonding between the oxygen atoms of POMs and functional groups of the peptides can also play a role in this first step. Then, these clusters can further interact *via* non-covalent van der Waals forces and hydrophobic interactions to form supramolecular structures such as the spheres shown in Fig. 5 (right, purple circles).

The two-step assembly of cationic peptides and POMs was described more in depth by Zhang *et al.* in 2015.^31^ They used isothermal titration calorimetry (ITC) to decipher the binding process in ionic POM–peptide hybrids formed by europium-containing POMs, {EuSiW_10_Mo} and {EuW_10_}, and arginine- and lysine-rich peptides (derived from HPV capsid proteins). In the first step, POMs would initially induce the disruption of the peptide assembly and then form strip-like primary clusters, which further assemble during step II into macromolecular structures, mainly nanospheres, driven by hydrogen-bonding interactions, van der Waals forces, *etc.* This simple yet promising assembly mechanism has now been used routinely to develop a range of class I POM–peptide hybrid materials, with different properties and applications (section 4).

**Fig. 6:**
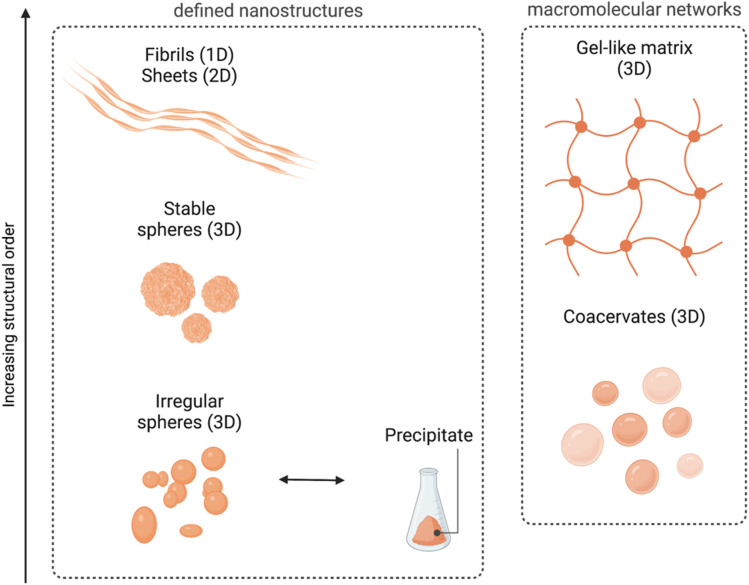
Overview of the different morphologies found for ionic POM–peptide hybrids, either as defined nanostructures or as macromolecular networks.

Even if such nanospheres are the most commonly observed POM–peptide assemblies, other well-defined structural morphologies, such as fibrils or macromolecular networks, have also been reported (Fig. 6). For example, the heteropolyacid {HSiW_12_} could direct the self-assembly of short facial-like peptides, which combine hydrophobic and hydrophilic residues, into well-defined nanofibres of *ca.* 13 nm of diameter.^27^ The authors described the inner structure of the fibres with the aid of high-resolution TEM images, which revealed the composition of the core of the fibre with four peptide and four polyoxometalate units, bound by a combination of hydrophobic interactions and π–π stacking between the central peptides and electrostatic interactions between the lysine residues of the peptide and the POMs (see Fig. 4 for a schematic representation of the fibres). Two additional peptide units, bound again by hydrophobic interactions and π–π stacking to the external peptides of the core, form the shell of the fibre. These shell peptides reduce the surface hydrophobicity of the fibres increasing their stability in water, resulting as well in a positively charged surface, property that can be exploited for antimicrobial applications (refer to section 4.3.1). Since this pioneering work, Wen Li and co-workers have studied the driving forces behind the formation of different macromolecular structures and how to balance these forces and engineer the components to create specific structures, from stable nanofibres (1D)^27,32^ to nanospheres (3D),^33^ or nanosheets (2D).^34,35^ Using different types of POMs and peptides, they observed that the three main factors for the number of dimensions of the assemblies are: (i) the steric hindrance, directly related to the size of the POM, (ii) the presence of aromatic or hydrophobic groups as well as cationic groups in the peptide, and (iii) the electrostatic repulsion produced by the shell peptides. Furthermore, self-assembly of the bioactive decavanadate {V_10_} anion with a cationic peptide produced helically twisted fibres whose rotation could be controlled by changing the chirality of the amino acids.^32^

Recently, Wen Li and co-workers have also explored the fabrication of 2D materials from POM–peptide hybrids. First, they could observe the formation of nanosheets upon UV radiation of a solution containing irregular nanospheres formed by a *trans*-azobenzene peptide and the POM {CoW_12_}.^34^ Indeed, it was the UV-induced transition from *trans*-to-*cis* conformation of the azobenzene side chains of the peptides which templated the transformation into *ca.* 6 nm-wide nanosheets. This transition was reversible, and the system returned into irregular spheres once the radiation was interrupted. From the knowledge obtained from this work, the same group decided to design the peptides using regular/non-synthetic amino acids which could spontaneously template the self-assembly into nanosheets without the need for an external source of energy.^35^ Indeed, thin nanosheets were obtained with different types of POMs, from the smallest Keggin-type {CoW_12_} to the Preyssler-type {P_5_W_30_}. Larger POMs such as the {Mo_132_} wheel were too large and unfavoured the stacking of the system. There is no doubt that controlling the self-assembly of POMs and (cationic) peptides presents an important potential to produce nanomaterials with defined nanostructures in different dimensions, which in turn can enhance their properties and amplify the possibilities of their applications.

Interestingly, all the ionic assembly processes reviewed so far have been produced in water and in a concentration range of 10 to 100 μM. Using other solvents as well as increasing the concentration of the components could change the rules in the assembly, for example triggering gel formation. Indeed, this is what Wen Li and co-workers observed when working with heteropolyacids – {HSiW_12_}, {HPW_12_} and {HPMo_10_V_2_} – and short lysine-rich peptides, which produced irregular nanospheres when mixed in water at concentrations of *ca.* 100 μM, whereas 3D network structures with gel properties were formed when combined in a mixture of EtOH/H_2_O at higher (100× more) concentrations. They also observed that the heteropolyacid played an important role triggering the gelation of the cationic peptides, since it provided the solubility needed in ethanol. Furthermore, the combination of basic and acidic amino acids in short and biocompatible peptides increased the adhesive properties of the 3D networks created by assembly with HPAs.^36^

In a middle point between the nanostructures found in solution and the gel-like macromolecular networks we can find the formation of coacervates. Coacervation implies a liquid–liquid phase separation process within colloidal dispersions and is nowadays an important method for encapsulation of special relevance in food and pharmaceutical industries among others.^37^ Wen Li and co-workers have recently explored the formation of complex coacervates containing Keggin-type POMs such as {SiW_11_}, {PW_9_} or {HPMo_10_V_2_} and single amino acids (histidine or arginine) or tripeptides, and how to modulate the transition from their fluid to a gel state by changing the pH or by addition of metal ions.^38–40^ In section 4.1, we report how the properties of such coacervates can be tuned to improve their applications as biocompatible adhesives.

Other examples of nanofibres and nanorods formed by assembly of peptides and POMs have been described in the literature with differentiating characteristics. The special case of Zhang *et al.*^41^ which used a tripeptide of formula Fmoc-FWK-NH_2_, that acts as nematic liquid crystal in acidic aqueous solution, *i.e.*, forming parallel filaments by self-assembly. Once aqueous solutions of POMs – {HPW_12_}, {BW_12_} and {V_10_} – were added to the liquid crystal solution, a hydrogel was formed. Electron microscopy images revealed that the hydrogels were formed by a 3D network of well-defined nanofibres of *ca.* 20 nm in diameter. In turn, these nanofibres were composed of the peptide filaments bound together by electrostatic interactions with the POMs. On the other hand, POMs have also been reported to provoke the disruption of nanostructures such as sheets to reorganise the assembly of a peptide-based polymer into nanorods with antimicrobial activity, as described in 2017 by Datta *et al.*^28^

It is worth mentioning the hybrids formed by assembling “virus-like particles” (VLPs) and POMs. VLPs have the same structure of the viral capsids and can be used in vaccines or diagnosis, for example. Yuqing Wu and co-workers explored the assembly of VLPs formed with peptide fragments of a capsid protein from different strains of the human papillomavirus (HPV) with two types of POMs: {EuW_10_} and {Mo_154_}.^42,43^ The authors used two different methods which provided in turn different results. One consisted of the co-assembly of the peptides and the POMs by incubation of the components and subsequent purification by dialysis. This protocol leads to the encapsulation of POMs inside the cavity of the VLPs. The post-assembly protocol allowed first the formation of the empty VLPs, which were then incubated in the presence of the POMs. This resulted in the attachment of the inorganic clusters to the surface of the VLPs. These different protocols, as well as the nature of the POM used, not only produce different types of assemblies but also tune their properties and therefore their applications as discussed in section 4.3.

Even though the examples of hybrids obtained *via* colloidal chemistry methods are so far the most predominant, some authors have followed alternative chemical synthetic methods, including molecular crystallisation, to produce new hybrids. Furthermore, there is extensive literature in the synthesis and characterisation of POM-based complexes that use amino acids and short peptides as ligands, which has been covered in different review articles.^29,44,45^ Since the seminal work of Crans and co-authors,^46,47^ the study of these structures and how POMs and amino acids and peptides interact has paved the way to a better understanding of the interaction of POMs and proteins, especially relevant for the development of biological applications.

For example, in the recent years, the group of Kessler has published two studies on the interaction of Keggin anions of the formula [PM_12_O_40_]^3−^ (M = Mo, W) with the dipeptide, tripeptide and tetrapeptide of Gly (GlyGly, GlyGlyGly and GlyGlyGlyGly) and the amino acid arginine, studying both the structure and behaviour of these complexes both in solution and in solid state.^48,49^ X-ray crystallography results revealed how the different polarity of the M=O bonds in the Keggin (Mo=O < W=O) influenced the mode of interaction between the peptides and the POM clusters. While electrostatic interactions dominate for {PMo_12_}, hydrogen bonding gains importance in the case of {PW_12_}. Furthermore, they observed that the longer the peptide chains the higher the interaction between the peptides and the POMs, producing more hydrophobic molecules and improving the stabilisation of the POMs from hydrolysis, even in diluted solutions. The Cronin group has explored the use of amino acid and oligopeptides as ligands to construct chiral frameworks based on Molybdenum Blue (MB) clusters which include lanthanide ions as symmetry breakers.^50,51^ Metal-coordination and H-bond interactions govern the self-assembly into nanowheels. These structures could be of great interest for the further development of inorganic proteins and artificial membrane channels.

### 2.2. Ternary hybrids

**Fig. 7:**
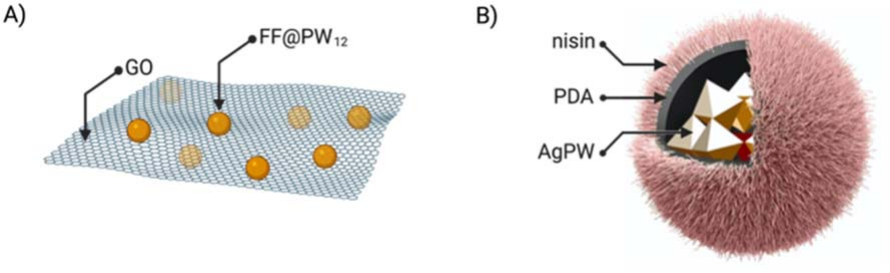
Ternary composites of ionic POM–peptide hybrids: (A) FF@PW_12_@GO and (B) AgPW@PDA@nisin, adapted from ref. 52 with permission, Elsevier, copyright 2020.

If the combination of just two different components offers enormous possibilities for creating new structures and materials, introducing a third component could expand even more the scope of these new materials once the possible setbacks would be overcome. For example, introducing a third element can destabilise the so-far binary assembly – which in some cases has been used as an advantage to have new applications.^52^ The first example of ternary hybrids incorporated a diphenylalanine peptide (FF), the heteropolyacid {HPW_12_} and graphene oxide (GO).^23^ Firstly, they produced the co-assembly FF@PW_12_ by the previously seen two-steps assembly method. GO has a laminar structure and therefore, when mixed with the peptide and POM, the FF@PW_12_ hybrid would maintain its spherical structure adhering to the surface of GO (Fig. 7A). Another example of ternary hybrids are the nanoflowers formed by the Keggin-type POM {Ag_3_PW_12_}, polydopamine (PDA) and the FDA-approved antibacterial peptide nisin. In this case, the composites AgPW@PDA@nisin were obtained by a first encapsulation of the POM by *in situ* dopamine polymerisation and followed by the immobilisation of the peptide on the surface of the POM–PDA hybrid (Fig. 7B).^52^

## 3. Covalent POM–peptide hybrids

The organo-functionalisation of polyoxometalates (POMs) is often a challenge because of their scarce solubility in organic solvents and limited stability towards acidic or basic media. POMs are oxidising agents that narrow the pool of available organic building blocks requiring robust organic chemistry. These problems, however, can be circumvented by tuning the synthetic approach, for instance, the solubility of POMs in organic solvents can be achieved employing different counterions, such as tetraalkylammonium cations.^53^

POMs can be considered as carboxylic acid surrogates, which can act as mild nucleophiles that can undergo activation for example *via* carbodiimides,^54^ esterification-like reactions,^55^ and cation chelation.^7^ The nucleophilicity of POMs comes from the free hydroxyl groups at the surface of the metallic cluster, in this way lacunary species might be better nucleophiles than plenary structures, due to the exposed available –OH groups at the surface. For instance, plenary {PW_12_} cannot chelate M^2+^ cations while {PW_11_} can.

**Fig. 8:**
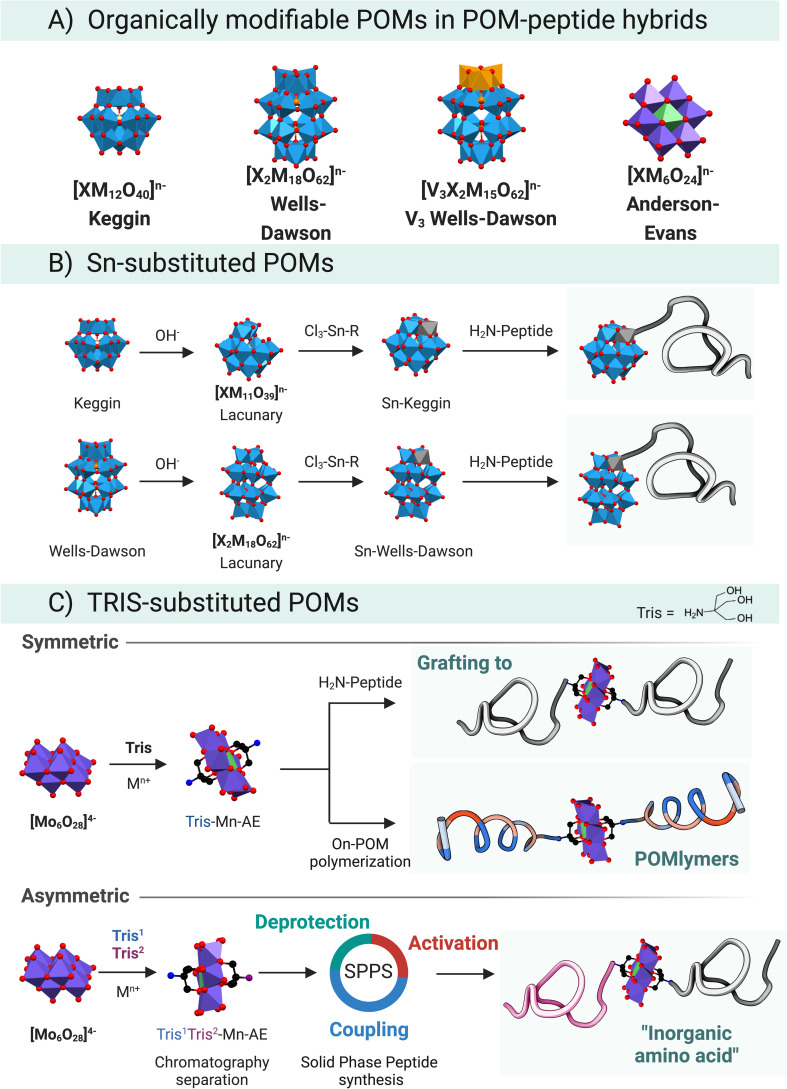
Overview of the different synthetic routes currently available to produce covalent POM–peptide hybrids. (A) Structures of the POMs conjugated with peptides in the literature. (B) Tin-strategy has been described for lacunary Keggin and Wells-Dawson POMs. (C) TRIS-functionalization strategy. Above is described the symmetric derivatization of a Mn–AE POM *via* direct covalente conjugation or by the on-POM ring opening polymerization approach. Down is depicted how to prepared an asymmetric Mn–AE and its incorporation in a SPPS workflow as inorganic amino acid.

Even though many covalent functionalisation strategies have been described for POMs,^56^ only two of them have been successfully applied to peptide–POM hybrids (Fig. 8A). The tin strategy, which has been explored in depth by Lacôte and co-workers,^57–60^ employs lacunary Wells–Dawson {P_2_W_17_} or Keggin {PW_11_}/{SiW_11_} anions (Fig. 8B). The lacunary structure readily reacts with a tin-based organometallic compound that acts as an electrophilic centre. The selected organo-tin is the handle for subsequent chemistry. Lâcote and co-workers used an organotin derivative of propanoic acid, which underwent lactonisation in the presence of triethylamine resulting in a stannalactone that involved the tin and a hydroxyl group of the lacuna.^59,61^

The prolific use of the TRIS-functionalisation strategy to prepare hybrid POMs has also facilitated the development of POM–peptide conjugates (Fig. 8C). Tris(hydroxymethyl)aminomethane (TRIS) coupling reaction can be understood as an esterification between the acidic –OH at the surface of the POM and the hydroxyl groups of the TRIS. The first reported synthesis of a TRIS-functionalised POM goes back to a report by Zubieta and Chen where a polyoxovanadate (POV) was successfully derivatised with TRIS-NO_2_.^62^ This organic derivatisation permitted isolating an otherwise hydrolytically unstable POV, highlighting one important role of an organic functionalisation. Thereafter, the seminal works of Hasenknopf *et al.*^63^ and Song *et al.*,^64^ on the preparation of TRIS-based Anderson–Evans (AE) POMs truly cemented the TRIS-functionalisation strategy as a convenient strategy to develop organo-functionalised POMs. Moreover, the AE scaffold has been derivatised with different chemical functional groups and metallic heteroatom centres *e.g.*, Fe^3+^, Mn^3+^, Al^3+^ to name just those most widespread. The Parac-Vogt group has recently studied the stability of Mn–AE hybrids, which will aid to strengthen the bases for the AE chemistry.^65^

POVs behave similarly to the AE scaffold and readily react with TRIS derivatives yielding a large pool of combinations. From the chemical point of view, the work of Hill and co-workers has focused on the esterification reaction that takes place in the V_3_-substituted phosphotungstate Wells–Dawson, [P_2_V_3_W_15_O_58_]^5−^.^55^ For a more comprehensive overview, please refer to a recent review by Parac-Vogt and co-workers, in which the state-of-the-art in POM post-functionalisation has been summarised, highlighting the key design and structural features that permit the discovery of new hybrid-POM platforms.^8^

Research by Cronin, Song, and co-workers^64,66^ paved the way towards the synthesis of complex asymmetric Mn–AE hybrids, for which two main approaches can be identified. The first one relies on differences between the crystallisation kinetics of symmetric *vs.* asymmetric hybrids,^64^ which is limited by the substituents in the TRIS moiety. The second approach starts from a mixture of two TRIS-R molecules, which react with the polyoxomolybdate to yield a mixture of products that can be separated by reverse-phase chromatography. In a variation on this strategy, a protected TRIS-based ligand ensures that, after separation by RP-HPLC, an asymmetric scaffold can be obtained through cleavage of the protecting group (Fig. 8C, asymmetric).^66^

Once the organic functional groups have been introduced, derivatised POMs can be used as conventional organic building blocks whose reactivity depends on the anchored functional group.

Regarding the tin strategy, two main approaches have been developed for the preparation of peptide-POM hybrids. Firstly, nucleophilic ring-opening of the stannalactone by the N-terminus of a peptide represents a flexible strategy.^59,60^ This strategy has been used for the kinetic resolution of α_1_-{P_2_W_17_} enantiomers in a series of very instructive publications which are beyond this Perspectives article.^57,59,60,67^ Secondly, Nikoloudakis *et al.*^68^ started with a tailor-made bisaromatic-tin linker with a carboxylic acid. Such acids can be coupled *via* the mixed anhydride method with the N-terminus of a Phe–Phe peptide.

**Fig. 9:**
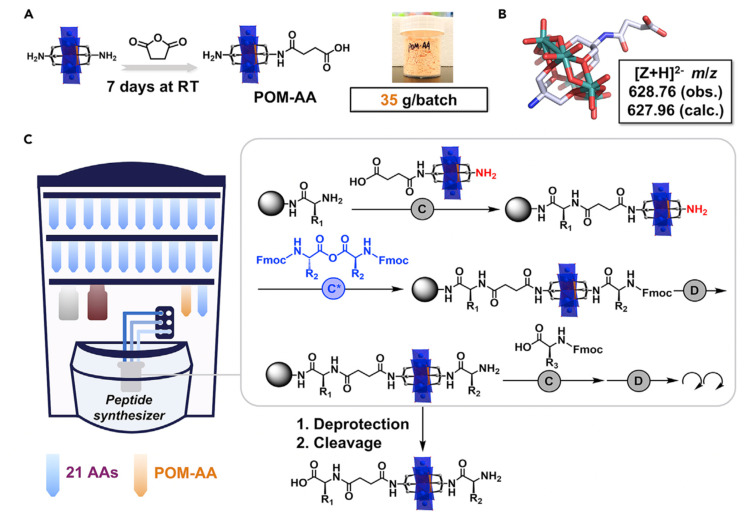
Schematic representation of POM–AA and POM–peptide synthesis procedure: (A) design and fabrication of POM–amino acid; (B) single-crystal structure of the POM–amino acid; (C) representation of the automated synthesis of POM–peptide on a peptide synthesiser. The grey box highlights the fundamental unit operations required in each step. Abbreviations = (C) standard coupling (4 eq. amino acid, 4.4 eq. DIC, and 4.4 eq. HOBt, 75 °C); (D) standard deprotection (20% piperidine in DMF, room temperature [RT]); (C*) anhydride coupling (4 eq. amino acid anhydride, 2.5 eq. DIPEA, 75 °C).^73^ Reproduced with Permission from Elsevier.

The most developed route to produce covalent POM–peptide hybrids is based on amino chemistry, with either the symmetric or asymmetric Mn–AE (Fig. 8C). For instance, Carraro^69,70^ and Cronin^71–73^ reported the preparation of a carboxylic acid derivative from TRIS that can be activated to couple a peptide *via* the N-terminus. Cronin *et al.*,^72,73^ reported the use of a POM as an inorganic amino acid in the preparation of an asymmetric Mn–AE containing one activated carboxylic acid, as well as a protected amino group. Such a POM derivative can be incorporated into the SPPS workflow: once the POM–amino acid has been reacted with the N-terminus of a crescent peptide the amino TRIS group can be deprotected and reacted with an activated amino acid to resume the peptide synthesis. Very recently, Cronin *et al.*^73^ reported a major improvement in the preparation of the *inorganic amino acid*. The route consists of the equimolecular reaction of Mn–AE with succinic anhydride over seven days. Surprisingly, after a simple ether precipitation NH_2_–Mn–AE–COOH was obtained and incorporated into a robotic SPPS set-up (Fig. 9). This strategy is inherently simple and easy to implement in a wide range of peptide sequences which facilitates complex studies as demonstrated by Cronin *et al.*^73^

**Fig. 10:**
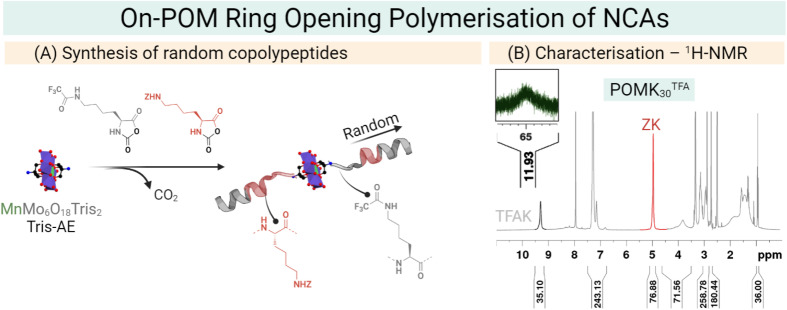
(A) General scheme for the synthesis of POMlymers from random copolypeptides by the on-POM ring opening polymerisation (ROP) of NCAs – lysine trifluoroacetate NCA (grey, TFA K) and benzyloxycarbonyl lysine NCA (ZK NCA). (B) ^1^H-NMR characterisation of POMK_30_^TFA^ which stands for 30 TFAK residues.

Our group has developed a new synthetic approach that uses the symmetric NH_2_–Mn–AE–NH_2_ as an initiator for the preparation of polypeptides as antimicrobial materials. We employed an amino-derivatised Mn–AE to initiate the ring-opening polymerisation (ROP) of an amino acid *N*-carboxyanhydride (NCA). In this *on-POM polymerisation*, the polymer grows directly from the POM centre which allows the preparation of hybrid materials in only one reaction step. The *on-POM polymerisation* strategy represents a modular and facile opportunity to explore the role of the covalent linkage in these hybrids (Fig. 10).^26^ Plus, ROP of amino acid NCAs is a living polymerisation and, therefore, the *on-POM polymerisation* strategy can give access to more complex structures as block copolypeptides.

## 4. Properties and applications

**Fig. 11:**
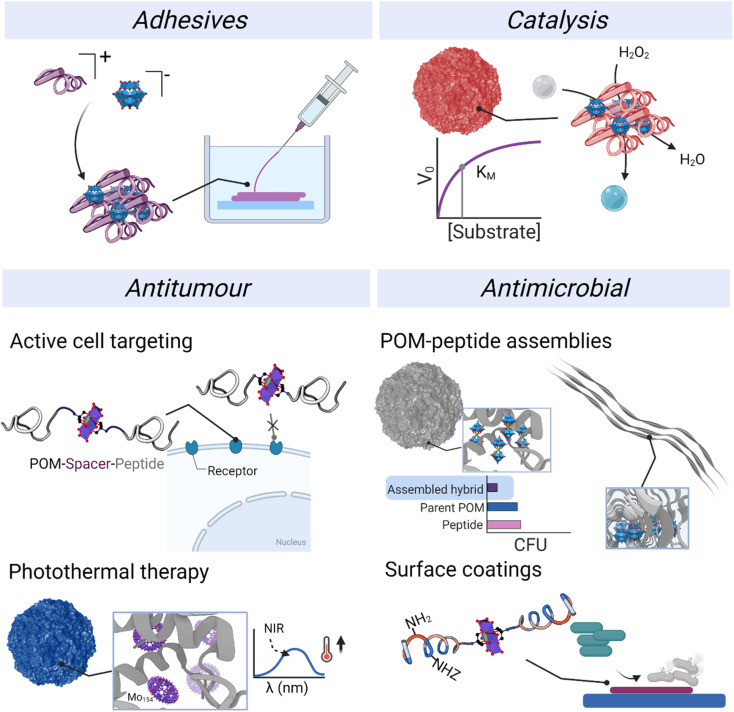
Overview of the most common applications explored for POM–peptide hybrids.

The development of applications has been carried out preferentially with ionic assemblies, which could, at least in part, be because their simple synthesis allows a faster exploration of broader chemical space and offers greater potential to obtain gram-scale quantities for application testing. In contrast to POM–peptide ionic hybrids, class II covalent hybrid materials typically provide smaller quantities of materials. Among the different applications published to date, we have divided the reported contributions into adhesives, catalysis, and biological applications (Fig. 11). In addition, we highlight the role of both component and how the application can benefit from an informed choice of the components and the conjugation or complexation strategy.

### 4.1. Adhesives

Peptides are useful materials for promoting adhesion to surfaces through diverse functionalities like thiols, amines, carboxylic acids or phenols. Indeed, mussels employ adhesive peptides, likely secreted as coacervates, to stick to wet surfaces.^74,75^ The combination of these peptides – typically containing a mixture of charged residues and chelating ones – and POMs represent a versatile strategy to improve adhesion properties.

Li's group have thoroughly explored this approach and proposed several designs for preparing underwater adhesives.^36,38,39,76,77^ The supramolecular polymerisation of the peptide produces coacervates, *i.e.* liquid–liquid droplets, that promote this adhesion phenomenon. Here, the POM is crucial to screen the ionic charges of the peptide, the condition required for the phase transition. More recently, they have introduced reversible adhesives based on non-covalent chemistry. For instance, the incorporation of histidine (His) allows pH responsiveness since at pH > p*K*_aH_ ≈ 6.0, His residue loses the positive charge thus disrupting the coacervate. What is more, His coordination to transition metal ions like Co^2+^ increases crosslinking density thus favouring both adhesive and mechanical properties.^39^ More recently, they used glutathione (GSH) as a redox-responsive peptide. At low pH, oxidised GSH co-assembled in a coacervate with the POM yielding an underwater adhesive. However, when immersed in a reducing solution the disulphide breaks which disrupts the coacervate, losing the adhesive properties.^77^

**Fig. 12:**
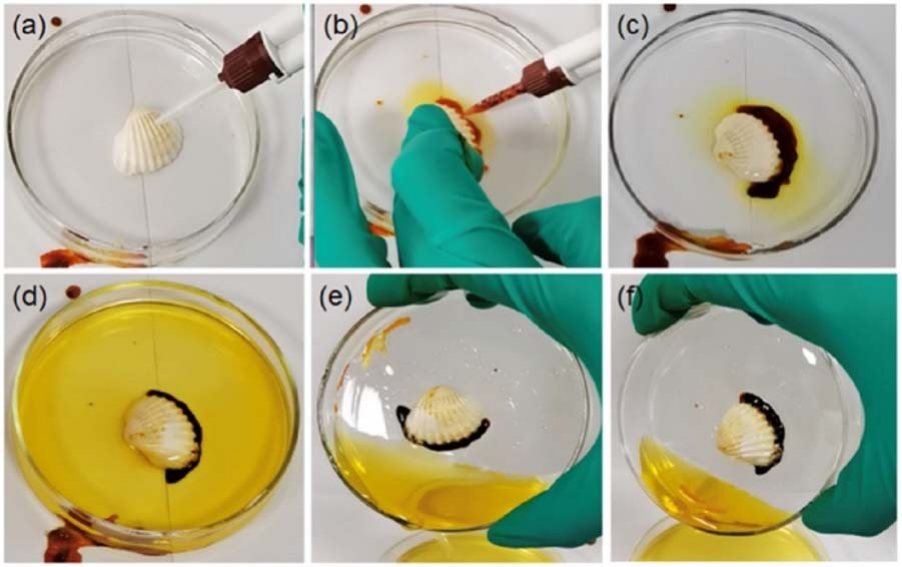
Photographs showing the curing and adhesion efficacy of the peptide/POM coacervates system based on {BW_12_} and {HPMo_10_V_2_} POMs. (a) A shell is immersed in water in a watch glass and (b) the coacervates are injected simultaneously. (c) and (d) Crosslinking of the shell takes place spontaneously. (e) and (f) Tilting tests show the effectiveness of the adhesion. Reprinted with permission from ref. 76. Copyright 2021 American Chemical Society.

Recently, the same group reported a unique strategy to prepare POM–peptide underwater adhesive hydrogels. The approach relies on mixing two redox-complementary peptide/polyoxometalate (POM) coacervates, one of them containing an oxidative POM – H_5_PMo_10_V_2_O_40_ {HPMo_10_V_2_} – and a cationic peptide, and the other formed by a reductive POM – K_5_BW_12_O_40_ {BW_12_} – and a cysteine-containing peptide. Upon mixing, PMoV oxidises cysteine triggering gelation even under water. The crosslinked hydrogel presents higher strength and better cohesion properties, especially when compared to the parent coacervates (Fig. 12).^76^

An interesting avenue for these materials would be to couple the oxidation of the peptide with the redox properties of the own POM. In this way, heteropolyblues (reduced POMs) could be prepared. Possibly, these reduced POMs could play an important role in the adhesion, since as we already mentioned, reduction induces assembly of the clusters. Other applications in the field of engineering and construction could be found in the production of anticorrosion and redox-active adhesives. This application is exemplary of how the design and synthesis of these materials have the greatest impact on the application, as they allow tuning the mechanical properties, the kinetics and the reversibility of the adhesion. Besides ionic interactions – fundamental in these materials – the redox activity, pH responsiveness and metal coordination ability offered by the building blocks are essential for this fine-tuning.

### 4.2. Catalysis

POMs are widely employed as catalysts since their redox properties can be derived from their formula and structure, thus allowing fine-tuning of the catalytic properties.^78,79^ We can find extensive literature regarding the use of POM–organic composites in catalysis, both with covalent and ionic hybrids.^80,81^ For instance, it is worth remarking on the scheme proposed by Wu and co-workers which uses the giant polyoxomolybdate {Mo_154_} functionalised with a cationic cyclodextrin (CDC) for cyclohexene oxidation. This system provides several advantages compared to the individual components. First, the oxidation catalyst {Mo_154_} is more stable when anchored to the CDC, which increases the reaction yield. Second, the CDC provides selectivity *via* the sieving effect through host–guest interactions. CDC preferentially allocates cyclohexene rather than other larger molecules. Finally, the catalysis process is enhanced upon NIR irradiation due to the photothermal properties of {Mo_154_} that produces a local temperature increase.^81^

Despite the numerous examples and applications in catalysis of POM–organic-based materials, POM–peptide contributions remain low but suppose great potential in the field of biocatalysis. In most of the examples, POM–peptide hybrids can be thought of as artificial metalloenzymes where the POM is the cofactor providing function.

The ternary hybrid material FF@PW_12_@GO reported by Ma *et al.* (Fig. 7A) displays peroxidase activity superior to the parent POM, the Keggin anion {PW_12_}. The hydrophobic environment created by the peptide favours substrate accumulation, thus increasing the selectivity even compared to the wild-type HRP.^23^

**Fig. 13:**
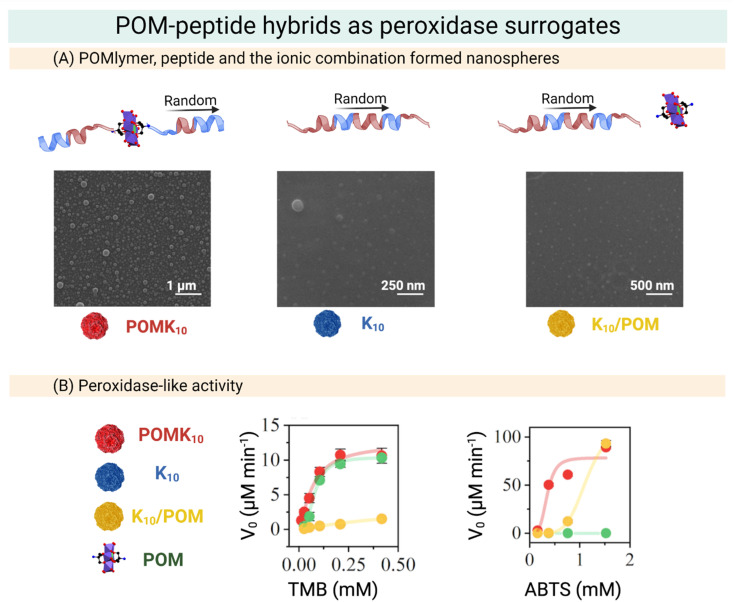
Overview of the preparation of POM–peptide hybrids as peroxidase surrogates. (A) SEM micrographs of POMK_10_, K_10_ and K_10_/POM, where POM stands for Mn–AE and K the number of lysine residues. (B) Steady state assays of POM–peptide hybrids in the oxidation of organic molecules. Left: Initial velocity of TMB oxidation at different concentrations. Right: Initial velocity of ABTS oxidation at different concentrations. Reproduced with permission from ref. 22, Royal Society of Chemistry, Copyright 2022.

Our group has developed POM–peptide hybrids (POMlymers, Fig. 13) as peroxidase mimics. We evaluated whether the POM–peptide connectivity influences the redox properties of the hybrid observing that nano assemblies of the covalent hybrid not only preserve the redox properties of the parent POM (Mn–AE) but also widen the scope of the reaction and can catalyse the oxidation of an anionic substrate. Interestingly, covalent POMlymer nanoparticles lost their catalytic effect when disassembled, pointing out the importance of generating sites for catalysis, similar to catalytic pockets in Nature (Fig. 13).^22^

### 4.3. Biological applications

The frontier research of biological applications of POMs is a rich field in which researchers from different research areas come together to harness the bioactivity of POMs and understand their mechanisms of action.^82^ While POMs have been extensively described as versatile molecules that display both antitumor and antimicrobial properties,^24,83^ the mechanisms of action and key properties behind their activity are still not fully understood. So far, their redox activity and their interaction with biomolecules and cell membranes are considered the main factors.^24^ Indeed, POMs unite distinctive properties in a single molecule: their size, net charge and charge density, activity from specific atoms and redox activity. All these properties provide great opportunities for developing new bioactive materials. At the same time, the structural composition and therefore chemistry of POMs can vary significantly under biological conditions involving changes in pH and ionic strength. Therefore, stabilising POMs inside polypeptide nanostructures represents a potential strategy for preserving the inherent properties of the POM and at the same time, the combination with already bioactive peptides could result in additive or synergistic effects. In this section, different applications of POM–peptide hybrids in biology will be revised, from antimicrobial to virus detection.

#### 4.3.1. Antimicrobial

One of the first examples of antimicrobial POM–peptide materials was described by W. Li and co-workers. They used the combination of a facial-amphiphilic positive peptide – without antimicrobial activity – with a Keggin heteropolyacid anion {HSiW_12_} to prepare an ionic assembly with better antimicrobial properties against *Escherichia coli* (*E. coli*) than the individual components.^27^ In 2017, Datta *et al.*^28^ developed a protein-like polymer that ionically co-assembled with {HPW_12_} creating nanorods active against both *E. coli* – model for Gram negative bacteria – and *Bacillus subtilis* (*B. sub*) – model for Gram positive bacteria. In these contributions, the assembly of cationic peptides with anionic POMs creates a material with a positive net charge and tubular structure which permits a great active surface/area of interaction with bacteria. This feature is important since the principal mechanism of action relies on the disruption of the bacteria's membrane. Besides this contact mechanism, in the work of Datta *et al.* an additional pathway of killing is described. Once in the cell, the POM is released from the nano-assembly, which produces oxidative stress leading to DNA damage and finally apoptosis. Therefore, the POM could be acting not only as a passive crosslinker but also as an inducer of oxidative damage.^28^

**Fig. 14:**
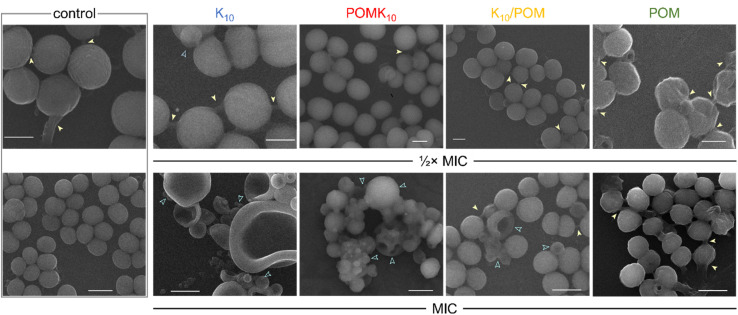
Scanning electron microscopy (SEM) images of *S. epidermidis* without treatment (control) and after incubation with peptide K_10_ (blue), covalent hybrid POMK_10_ (red), ionic hybrid K_10_/POM (yellow) and parent POM (green) at their 1/2× MIC (scale bar = 500 nm) and MIC (scale bar = 1 μm). Yellow arrows indicate biofilm matrix, blue outlined arrows show cell deformation or damage. Reproduced with permission from ref. 22, Royal Society of Chemistry, Copyright 2022.

We have been working on POM–peptide covalent conjugates for antimicrobial applications. In our first contribution, we observed that covalent POMlymers could be coated on surfaces to prevent *B. sub* biofilm formation.^26^ Then we explored how self-assembly and POM connectivity (covalent or ionically linked) affected the antimicrobial properties against the Gram-positive *Staphylococcus epidermidis*. As seen in catalysis studies, covalent and ionic POMlymers differed in their *in vitro* behaviour. In this case, the ionic nanoparticles displayed the same minimal inhibitory concentration (MIC) as the free polypeptide (62.5 μg mL^−1^), whereas the covalent assembly had a higher MIC (125 μg mL^−1^). An interesting feature was that the addition of the parent POM did not enhance the antimicrobial activity. When we evaluated the oxidative stress response of the bacteria, the intracellular ROS production of the ionic assembly was commensurate with that of the parent POM. In both cases, ROS production stimulated the biofilm proliferation as a bacterial defence mechanism compared to the controls – treated with the peptide or untreated (Fig. 14). On the other hand, the covalent nanoparticles induced only mild oxidative stress and desired reduction of biofilm as compared to the controls. This work shows that the POM is released from the ionic assembly leading to two individual components, the POM and the free peptide, highlighting the importance of selecting the correct conjugation/complexation approach. In contrast, the covalent derivatives benefit from the combination of antimicrobial peptide and POM.^22^

A similar phenomenon was observed by L. Wu and co-workers who employed virus-like particles (VLPs) containing a human papillomavirus (HPV) capsid peptide (L1-p) with {EuW_10_}. Encapsulation of the POM inside the cavity of L1-p VLPs lead to an increased stability of the VLPs compared to POM-free peptide assembly, which could improve protein vaccines in the future. Although the empty L1-P VLPs didn't have any effect on bacterial growth, the L1-P VLPs containing POM exhibited higher antibacterial activity than the POM alone. In contrast, when the POM was decorating the surface in VLPs@EuW_10_, the system lost efficacy. As the POM is attached to the VLP surface, it is quickly released, yielding two individual entities – {EuW_10_} and L1-p – with low antimicrobial activity.^42^

Employing bioactive POMs and peptides could significantly increase the antimicrobial properties of the resulting hybrid. For example, the group of Qi co-assembled {AgPW_12_} with an antimicrobial peptide (nisin) in a polydopamine nanocapsule (Fig. 7B).^52^ The combination effectively killed *Staphylococcus aureus via* membrane disruption. It is worth mentioning that *Staphylococcus aureus* infections are a serious threat in hospital-acquired infections due to the extensive drug resistant strains (Fig. 16).

**Fig. 15:**
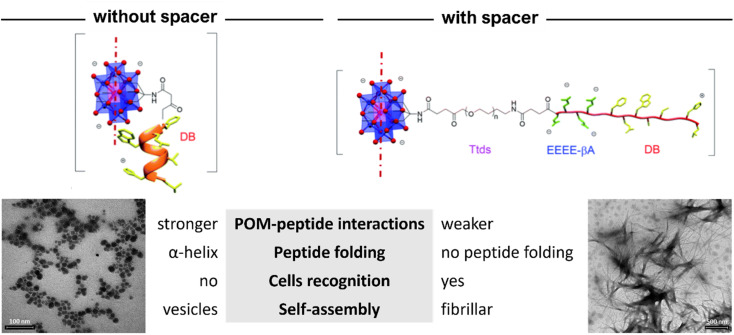
Structural differences between the hybrids of POM–(demobensin-1)_2_ without and with spacer between the Mn–Anderson–Evans POM and the peptide. Adapted with permission from ref. 70, Royal Society of Chemistry, copyright 2021.

**Fig. 16:**
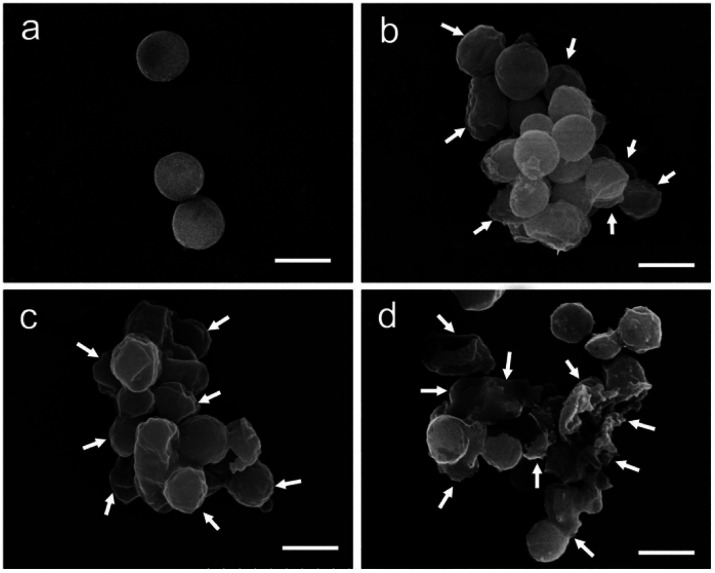
Damage on *Staphylococcus aereus* membrane produced by treatment with AgPW@PDA@nisin at its MIC (4 μg mL^−1^) for 0 h (a), 6 h (b), 12 h (c) and 24 h (d). The white arrows show the wrinkled and broken membrane structure. Scale bar: 500 nm. Reprinted with permission from ref. 52 Copyright 2020 Elsevier.

There is great potential in the research and development of new POM–peptide hybrids with antimicrobial properties. The final structure and charge density over the surface are one of the key parameters for improving their activity, by increasing the interaction with bacteria. Additional features such as redox activity or antibiofilm activity could be of great interest for these materials as well. Indeed, the production of biofilm matrix has been connected to mechanisms of antimicrobial resistance and has an important role in serious infectious diseases.^84^

The hybrids could also be designed as releasing agents of active molecules, for example, preventing hydrolysis of AMPs or POMs. Another interesting characteristic to consider in the design of antimicrobial POM–peptide hybrids would be its physical application which, for example, could be tuned to create coatings, especially relevant in the medical devices industries. As seen in the previous section, peptides can provide adhesive properties and contribute to better adhesion of the hybrid to the material to be coated. Most of the examples of POM–peptide hybrids studied as antimicrobial materials correspond to ionic hybrids. However, covalent hybrids could deliver advantages – for example antibiofilm activity – antibiofilm activity be of importance for generating the physical properties to be applied as coatings, for example.^22^

#### 4.3.2. Anticancer

POM–peptide hybrids have been designed for cancer treatment, for example, Carraro *et al.* developed a covalently linked peptide (demobensin) to Mn–AE that self-assembled in water solution.^69^ This peptide does not exhibit any secondary structure and has been used in targeted cancer therapy because its receptors are overexpressed in some types of cancer such as colon, lung, or prostate. They found that when grafted to the POM, the IC_50_ towards HeLa cells (cancer cells) decreased compared to the IC_50_ of the peptide, which correlated well with the intrinsic toxicity of the POM. However, the interaction with cells was not specific since when the authors used cells that did not overexpress the peptide receptor and the same toxicity was observed. In other words, the POM–peptide hybrids were entering in a non-selective fashion. The authors attributed this effect to the peptide folding into an alpha-helix, induced by the strong interaction with the POM, and the self-assembly of the materials into vesicles (Fig. 15). This is a key aspect to bear in mind since molecular recognition and thus bioactivity is directly related to structure. In a recent publication, the same group reported the incorporation of a spacer between the POM and the bioactive peptide to favour biomolecular recognition. By adding a small PEG-like chain spacer (Tdts) and an anionic poly(glutamic acid) sequence, which favours electrostatic repulsion between the POM and the peptide, Carraro *et al.* successfully improved the therapy in HeLa cells compared to the hybrids without the spacer.^70^

POMs can also be exploited as an active part of the assembly, for example, as we already commented, L. Wu and co-workers used a soybean antioxidant peptide to protect {PW_12_} when photoreduced (rPW_12_). rPW_12_ displays a UV-absorption band in the near-infrared (NIR) that produces heat when irradiated at 808 nm. POM–peptide nanoparticles displayed great biocompatibility; however, when cells were irradiated with the hybrids an abrupt decrease in viability was observed even when incubating 24 h in the air before irradiation. rPOM is active merely right after incubation since when the sample was incubated for 1 day in the air, no cell death was observed. This phenomenon highlights the importance of encapsulating the POM to avoid reoxidation, in this case, the oxidised POM is no longer photoactive.^85^ These nanohybrids prepared by Wu also displayed antimicrobial properties as previously discussed probably due to the concentration of positive charges at the surface of the nanoparticle, which lead to bacterial membrane disruption.

In line with this hypothesis, Sun and co-workers prepared VLP assemblies with {Mo_154_} as the metallic core and the HPV L1 capsid protein. They showed that VLP–Mo_154_ nanoassemblies are more stable than VLP assemblies, as previously observed with the EuW_10_–VLPs particles. In addition, these hybrid assemblies reduce cell viability in cancer cells (HeLa) compared with each component, and the effect is more pronounced after irradiation at 808 nm. The authors hypothesised that this enhancement was due to an increased cellular uptake that results in increased toxicity from the hybrid.^43^

#### 4.3.3. Other biomedical applications

Other biomedical applications have been explored for POM–peptide hybrid materials, including their use as biosensors or modulators of the aggregation of the amyloid-β (Aβ) peptide, implicated in the development of Alzheimer's disease (AD). A wide variety of POMs and POM-hybrids have been previously used as *in vitro* inhibitors or modulators of the aggregation of Aβ,^86–92^ for example by chelating metal ions such as Cu^2+^ which is involved in the aggregation of toxic Aβ oligomers and the production of ROS.^93^ There are four examples in the literature of POM–peptide hybrid materials designed to combat AD from different approaches. The ionic hybrids combine the derivative from the Aβ_15–20_ peptide (Ac-QKLVFF-NH_2_) – a fragment that can bind the full-length peptide and inhibit its aggregation – with a Wells–Dawson POM {P_2_CoW_17_}^94^ or a polyoxomolybdate (MoPOM),^95^ following the two-steps assembly previously described (Fig. 5). Both hybrids present better inhibitory activity than their parent components, reducing the final quantity of fibrils formed. In the case of covalent hybrids, Qu and co-workers have led the research, first with new POM hybrids with l/d-amino acids, of the formula (l/d-aa) {TRIS–MnMo_6_O_18_–TRIS} (l/d-aa).^96^ The authors showed how the chirality of the hybrids can have a positive impact on their activity and selectivity towards fibrillation inhibition. Even if all hybrids could prevent the aggregation of the Aβ peptide to fibrils, the (F)-modified POMs, and more specifically the (d-F)-POM showed stronger activity. In a new approach, which searches to mimic post-translational modification (PTM) of proteins and peptides, Gao *et al.*^97^ produced a Sn-functionalised Wells–Dawson POM with a thiazolidinethione unit which binds covalently to the Aβ peptide *via* an addition reaction to the Lys_16_ amino group. The new covalent hybrid formed by the {P_2_SnW_17_} and the Aβ peptide effectively hinders the aggregation capacity of the full-length peptide. Cronin *et al.* recently reported the influence of the position of Mn–AE within the peptide backbone using a similar approach as alanine scanning. The peptide sequence was derivatised from VLFF which consists of the hydrophobic domain responsible for aggregation. Incorporation of the POM targets the interaction with the positively charged domain in the amyloid peptide. When placed at the N-terminus (Mn–AE–VLFF), the POM–peptide hybrid slowed down amyloid fibres formation. This observation is quite interesting as an equally charged peptide containing three glutamic acid residues (EEEVLFF) performed worse than the hybrid peptide. Herein the interesting electronic properties of POMs, such as localised surface charge, may improve the performance.^73^

Another extensive field in the biomedical applications of POM–peptides hybrids explored by researchers has been the use of peptides derived from the human papillomavirus (HPV). The formation of most of these materials has been explained in section 2 and we will briefly explain their applications here. The most exploitable property of these hybrids is the fluorescence of the Eu-containing POMs upon which they are constructed: {EuW_10_}, {EuSiW_10_Mo} and {EuPW_11_}. Upon binding to the positively charged amino acids of the peptides, mainly fragments of the HPV-16 and HPV-18 capsid proteins rich in arginine and lysine, the solvating water around the Eu-POMs is displaced resulting in enhanced fluorescence.^31,98^

The same group further exploited these Eu-containing hybrids as luminescent sensors for the recognition of different biomolecules. Two different methods seem to govern the capacity to act as sensors. The first one relies on the quenching of the fluorescence *via* competitive recognition caused by the disintegration of the hybrid assembly upon addition of the biomolecule of interest.^99^ For this, the interaction between the POM and the peptide in the assembly should not be too tight to permit its dissemblance. The POM with the lowest charge, {EuW_10_} (−9), offered the optimal binding capacity in this case. In contrast, the second method searches an enhancement of the luminescence as sensing signal.^100^ First, a bi-assembly hybrid is formed with {EuW_10_} and GL-22, cationic peptide from the E6 oncoprotein of HPV-16. Upon addition of the biomolecule of interest, spermine – a biogenic amine – in this case, the fluorescence of the system is enhanced *via* synergistic binding of both cationic molecules. Furthermore, it was demonstrated that the EuW_10_/GL-22 assembly showed selectivity and a higher enhancement of the fluorescence. The antimicrobial and anticancer properties of hybrids formed with VLPs and POMs – {EuW_10_} and {Mo_154_} – has been previously detailed in sections 4.3.1 and 4.3.2. Introducing POMs into the VLPs assemblies plays another important role in the hybrid. Acting as “charge neutralisers”, assembly with POMs greatly increased the thermal, pH and storage stabilities of VLPs and could be of great significance in the development and use of protein-based vaccines.^42,100^ In 2021, a new hybrid based in the lanthanide {GdW_10_} POM and a food-derived peptide (KDHCHVTPY) was described to form rather small spherical aggregates, with diameter of *ca.* 4 nm. In this case, the lanthanide-based POM provides the properties needed to act as an agent contrast for both medical imaging techniques: magnetic resonance imaging (MRI) and computed X-ray tomography (CT).^101^

## 5. Conclusions and future outlook

While numerous synthetic design strategies for preparing hybrid POMs are now well known and utilised, we consider that research into POM–peptide hybrids still remains in its infancy. In this Perspectives article we have outlined the fundamental basis of this research area and have demonstrated that recently there has been an observable transition in the design paradigm. By addressing the literature from a multidisciplinary standpoint, we hope that this overview will be useful to researchers initiating in the field as well as for expert readers who require a snapshot of the current state-of-the-art in order to provide improved materials and technological solutions. Our conclusions and perspectives herein, point to several key challenges and opportunities for the area.

In broad terms, the chemical properties of both components have not yet been fully exploited to achieve their full potential to access hybrid materials with enhanced or synergistic properties. Most specifically, until now the inorganic POM component has served largely as a structural constituent, typically as an anchoring point for the peptide (class II hybrids) or as a supramolecular self-assembly tool (class I hybrids). And from a chemical point-of-view, where the bioactivity of such POM–peptides is concerned, POMs have often been mere spectators playing supporting roles to the properties of the peptide, *e.g.*, in the case of antimicrobial applications. However, their redox-active role is altogether more apparent in applications involving electron-transfer catalysis and as light-to-heat transducers for photothermal therapy. Herein lie opportunities for researchers interested in developing combinatorial libraries of class I and class II hybrids where the true potential of both POM and peptide can be maximised. In this respect, development of new covalent hybridisation approaches, such as *on-POM polymerisation* strategies and robotic SPPS, have the potential to facilitate access to POM–peptide structures. However, the Mn–Anderson–Evans – whether symmetrically or asymmetrically functionalised – has been the model POM anion for such developments, and researchers should aim to break free of this particular anion and opt for other moieties, such as the Wells–Dawson, to gain access to greater range of redox chemistry and catalytic activity. It is described in the literature that POM–peptide connectivity may play an important role in the biological activity. Consequently, it is of particular interest to develop alternative grafting approaches towards class II POM–peptide hybrids. A variety of different conjugation chemistries should be explored, for instance those based on organo-silanes or organo-phosphorus. Such hybrids would provide access to alternative POMs, such as the Keggin and Wells–Dawson anions, which showcase notoriously better redox properties than the often-employed Anderson–Evans motif. Varying the POM–peptide connectivity as well as the cluster size and charge may present interesting new avenues to fine tune biological responses in the final hybrids.

Furthermore, breakthroughs leading to substantial leaps for the field will arise from combining multifunctional POM and peptide components with more rigorous materials characterisation to understand and engineer next-generation materials that meet the particular needs of the given area of application, whether that be in catalysis, healthcare materials, or adhesives. Currently, however, there are still a limited number of examples where the fundamental physico-chemical properties of the hybrid material can be adapted or improved through fine tuning of the component parts. Herein lies the grand challenge for chemists and materials scientists: to chemically tailor POM–peptide materials to target specific end-applications. Consequently, the structure–activity relationships of new POM–peptides should be investigated in detail length. Currently, many of the examples of POM–peptide hybrids in the literature are one-off variants with applicable properties, but systematic investigation of varying POM and peptide components are rarely considered. In this respect, measurement standardisation may be required in order to compare interlaboratory data, which truly would help to advance the field.

Our analysis of the available literature on POM–peptide hybrids confirmed our premise that this is a rich and exciting area with a variety of different materials and applications offering challenging research opportunities for chemists and materials scientists aiming to develop functional materials. It is particularly relevant to those investigating in areas of robotic and microfluidic synthesis, as well as prospects for those working in the area of thin-film deposition, where such techniques could lead to new high-aspect ratio POM–peptide structures, controlled thickness levels, and tunable film compositions.

## Author contributions

All authors contributed equally to the conceptualisation, curation of literature, reviewing, and editing of this manuscript. H. S.-C. and E. A.-B. prepared all the figures and wrote the original draft.

## Conflicts of interest

There are no conflicts to declare.

## Supplementary Material
